# Natural selection on synonymous mutations in SARS-CoV-2 and the impact on estimating divergence time

**DOI:** 10.2217/fvl-2021-0078

**Published:** 2021-05-19

**Authors:** Yuanyuan Yu, Yan Li, Yu Dong, Xuekun Wang, Chunxiao Li, Wenqing Jiang

**Affiliations:** 1 ^1^Department of Anesthesiology, Qingdao Haici Hospital, Qingdao, Shandong, China; 2 ^2^Department of Cardiology, Qingdao Center Hospital, Qingdao, Shandong, China; 3 ^3^Department of Intervention, Qingdao Center Hospital, Qingdao, Shandong, China; 4 ^4^Department of Respiratory Diseases, Qingdao Haici Hospital, Qingdao, Shandong, China

**Keywords:** energy cost, natural selection, RNA structure, SARS-CoV-2, synonymous, tRNA

## Abstract

To adapt to human host environment, synonymous mutations in SARS-CoV-2 are shaped by tRNA selection, energy cost and RNA structure.

## Our knowledge of synonymous mutations

Synonymous mutations do not change the amino acid sequences. For a long time, synonymous mutations are regarded as neutral and silent mutations. In evolutionary biology, the estimation of divergence time between species requires a set of mutations with constant accumulation rate. The many deleterious missense mutations would be purged by purifying selection and thus are not suitable for measuring the divergence [1], whereas, synonymous mutations are free from natural selection and could provide a linear function to calculate the divergence time [2]. This notion has been well utilized to investigate the origin and evolution of SARS-CoV-2 by looking at the synonymous divergent sites between SARS-CoV-2 and RaTG13 [3].

However, in recent years, there is growing evidence demonstrating that synonymous mutations are not neutral. Researchers are getting aware that amino acid alterations (caused by missense mutations) are no longer the only feature to be subjected to natural selection. At molecular level, synonymous mutations could affect pre-mRNA splicing [4], tRNA availability [5], guanosine and cytidine (GC) content and codon usage [6], translational speed [7], protein extension [8], cost of energy and resources [9] and RNA structure/stability [10]. All these biological consequences of synonymous mutations may change the fitness and be exposed to natural selection. Beneficial and adaptive synonymous mutations are gradually getting fixed in a species while deleterious synonymous mutations are rapidly eliminated. In other words, the synonymous mutations do not accumulate with constant rate. This fact sheds doubt on the synonymous-based methodology of divergence estimation. Given the essentiality of clarifying the origin and evolution of SARS-CoV-2 under the current COVID-19 pandemic, more meticulous approaches should be considered to estimate the divergence.

In this article, we will first discuss the non-neutral property and the selection patterns on synonymous sites in SARS-CoV-2. We especially focus on the synonymous divergent sites between SARS-CoV-2 and RaTG13 because the latter is usually used as an outgroup sequence of SARS-CoV-2. Based on the many confounding factors disrupting the neutrality of synonymous mutations, we will try to propose a potential solution to the dilemma and wish to roughly estimate the genuine divergence time according to synonymous sites.

## SARS-CoV-2 needs to adapt to the host tRNA pool for efficient translation

Although synonymous codons encode the same amino acid, they could have different types of cognate tRNAs that decode the particular codon. The term tRNA adaptation index quantitatively describes the tRNA availability among synonymous codons [5]. Higher tRNA adaptation index correlates with faster translational speed and thus should be advantageous [11]. In humans, the C/G-ending synonymous codons usually have higher tRNA availability than the A/T-ending synonymous codons. For SARS-CoV-2, in order to adapt to the host tRNA pool and achieve higher translation efficiency, the virus should adjust its synonymous codon usage toward more C/G-ending codons which are favored by human.

There are hundreds of synonymous divergent sites between SARS-CoV-2 and RaTG13, mainly contributed by the two longest genes *ORF1AB* (618 sites) and *S* (215 sites) [3]. The synonymous mutations from RaTG13 to SARS-CoV-2 would be advantageous if they switch A/T to C/G, while the synonymous mutations from C/G to A/T would be deleterious. Indeed, the former class of mutations are more frequently observed between RaTG13 and SARS-CoV-2. Since the host of RaTG13 is not human, the distinct synonymous codon divergence between SARS-CoV-2 and RaTG13 simply indicates the selection force acting on synonymous mutations in SARS-CoV-2 that prompts the virus to adapt to the tRNA pool in human hosts.

To date, we know that the synonymous mutations in SARS-CoV-2 are not neutral and are already skewed by natural selection and; therefore, the divergence time based on synonymous sites should be re-evaluated.

## Energy cost also constrains the synonymous mutations

We have stressed the importance of considering tRNA availability and translation efficiency when judging the property of synonymous mutations in SARS-CoV-2. However, the cellular system is a balance between cost and efficiency. It is not worthy to slightly enhance the efficiency at extremely high costs. In order to rapidly proliferate, not only the efficiency but also the cost should be taken into account [9].

The biosynthesis of the four basic nucleotides consumes certain numbers of ATP molecules with the order of A > G > C > T [12]. Cheaper nucleotides (less ATP) are favored by natural selection. For synonymous codons, although C/G have the advantage of more efficient translation than A/T, they also cost more ATPs than T. Therefore, the selection pressure on synonymous mutations is a trade-off between energy cost and efficiency.

From the synonymous divergent sites between SARS-CoV-2 and RaTG13, it is hard to parse the selection on ATP cost because both species have the demand to lower the biosynthetic cost. However, from the polymorphic sites in SARS-CoV-2 population (derived mutations could be inferred from the outgroup RaTG13 sequence), one should observe higher derived allele frequencies (an indicator of positive selection) on the synonymous mutations that decrease the energy cost.

## Synonymous mutations alter the GC content & RNA structure

RNA structure also affects translation elongation and initiation [13]. C–G base pairs are more stable than A–T base pairs. Therefore, synonymous mutations that increase the GC content are likely to reinforce the RNA structure. It is known that highly structured RNAs are not favorable for efficient translation [14], then the mutations from A/T to C/G would suffer from the disadvantage of this structural effect. Again, the trade-off between RNA structure and tRNA availability should be balanced. Nevertheless, it seems that the structured regions only consist of a small part of the entire transcriptome as there is still the global trend that the synonymous mutations increasing the GC content are selectively advantageous.

## Extensive RNA modifications by the hosts

So far, we have discussed several biological features that are constrained by natural selection, making the synonymous mutations non-neutral. It reminds the researchers that the inference of divergence time from synonymous sites should be re-evaluated. However, this statement is based on the assumption that the synonymous mutations occur with constant rate (although, they accumulate with nonconstant rate due to natural selection). Next, we will show that even the occurrence of synonymous mutations does not conform to constant rates.

A broad range of species, from plants to animals, have the adenosine-to-inosine and cytidine-to-uridine deamination mechanisms to diversify their cellular RNAs [15]. When RNA viruses invade hosts, they frequently undergo RNA deamination by the host system, leading to A–G(I) and C–T(U) mismatches between the sequenced virus samples and the ancestral sequence. Intriguingly, the natural mutations in all organisms come from the DNA replication errors (for DNA organisms) or RNA reverse transcription/replication errors (for RNA viruses like SARS-CoV-2). The natural mutation rate is usually 1E-8 per nucleotide per generation. In contrast, the RNA deamination rate could be higher for orders of magnitudes. Evidence already shows that there is extensive RNA deamination in SARS-CoV-2 sequences [16,17]. More specifically, 87% of the synonymous divergent sites between SARS-CoV-2 and RaTG13 could be explained by RNA deamination rather than natural mutation, leading to overestimated divergence time [3]. This observation suggests that the synonymous variants in SARS-CoV-2 come from two resources with completely different mutation rates (natural replication errors vs RNA deamination), and also warns us that the prevalent deamination events should be considered when estimating the divergence time.

## Estimation of the genuine divergence time based on synonymous sites

We have shown that the synonymous mutations in SARS-CoV-2 do not occur under constant rate, and that they also undergo natural selection. Both features hinder the accurate estimation of divergence time based on synonymous sites.

We propose the potential approaches to correct the bias and estimate the genuine divergence time between SARS-CoV-2 and other related viruses.

- Avoid using the synonymous mutations that strongly alter the tRNA availability. For SARS-CoV-2, only keep the synonymous mutations between C and G or between A and T. These mutations are less affected by natural selection on tRNA availability.
- Discard synonymous mutations that dramatically change the ATP cost. As mentioned above, biosynthesis of adenosine costs most ATPs while thymidine costs least ATPs. Therefore, mutations between A and T are no longer considered.
- Discard A–G and C–T mutations that are potentially caused by deamination system. These two types of mutations are already excluded by the first criterion.

By this way, using the observed substitution on the remaining synonymous mutation sites and also considering the natural mutation rate, one could estimate the relatively accurate divergence time between SARS-CoV-2 and other viruses. We hope our summary and ideas could be interesting to the broad virology community as well as evolutionary biologists.
